# Inhibition‐directed multimodal imaging fusion patterns in adults with ADHD and its potential underlying “gene‐brain‐cognition” relationship

**DOI:** 10.1111/cns.13625

**Published:** 2021-03-16

**Authors:** Xiaojie Guo, Lu Liu, Tiantian Li, Qihua Zhao, Hui Li, Fang Huang, Yanfei Wang, Qiujin Qian, Qingjiu Cao, Yufeng Wang, Vince D. Calhoun, Jing Sui, Li Sun

**Affiliations:** ^1^ Peking University Sixth Hospital/Institute of Mental Health Beijing China; ^2^ National Clinical Research Center for Mental Disorders & Key Laboratory of Mental Health Ministry of Health (Peking University Beijing China; ^3^ Brainnetome Center and National Laboratory of Pattern Recognition Institute of Automation Chinese Academy of Sciences Beijing China; ^4^ University of Chinese Academy of Sciences Beijing China; ^5^ Tri‐institutional Center for Translational Research in Neuroimaging and Data Science (TReNDS) [Georgia State University, Georgia Institute of Technology, Emory University Atlanta GA USA; ^6^ CAS Center for Excellence in Brain Science Institute of Automation Chinese Academy of Sciences Beijing China

**Keywords:** adults with attention‐deficit/hyperactivity disorder, inhibition, multimodal MRI fusion, *NOS1* ex1f‐VNTR

## Abstract

**Aims:**

Inhibition deficits have been suggested to be a core cognitive impairment in attention‐deficit/hyperactivity disorder (ADHD). Exploring imaging patterns and the potential genetic components associated with inhibition deficits would definitely promote our understanding of the neuropathological mechanism of ADHD. This study aims to investigate the multimodal imaging fusion features related to inhibition deficits in adults with ADHD (aADHD) and to make an exploratory analysis of the role of inhibition‐related gene, *NOS1*, on those brain alterations.

**Methods:**

Specifically, multisite canonical correlation analysis with reference plus joint independent component analysis (MCCAR + jICA) was conducted to identify the joint co‐varying gray matter volume (GMV) and the functional connectivity (FC) features related to inhibition in 69 aADHD and 44 healthy controls. Then, mediation analysis was employed to detect the relationship among inhibition‐related imaging features, *NOS1* ex1f‐VNTR genotypes, and inhibition.

**Results:**

Inhibition‐directed multimodal imaging fusion patterns of aADHD were reduced GMV and FC in inhibition network and increased GMV and FC in default mode network. The results showed a significant indirect effect of *NOS1* ex1f‐VNTR on inhibition via FC component [effect size = −0.54 (SE = 0.29), 95% CI = −1.16 to −0.01]. In addition, the results indicated a significant indirect effect of GMV on the inhibition via FC component [effect size = 0.43 (SE = 0.23), 95% CI = 0.12 to 1.00].

**Conclusion:**

The findings suggested that reduced GMV and FC in inhibition network and increased GMV and FC in default mode network were jointly responsible for inhibition deficits in aADHD. Both the *NOS1* ex1f‐VNTR genotypes and GMV might influence the inhibition through the mediation effect of the aforementioned FC (*NOS1*/GMV^→FC→^Inhibition).

## Graphical abstract

The study suggested reduced gray matter volume (GMV) and functional connectivity (FC) in inhibition network and the increased GMV and FC in default mode network were jointly responsible for inhibition deficits in ADHD. Both the *NOS1* ex1f‐VNTR genotypes and GMV might influence the inhibition through the mediation effect of the aforementioned FC, which established a potential ‘gene‐brain structure/ function‐cognition’ pathway.

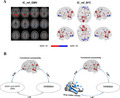

## 1 INTRODUCTION

Attention‐deficit/hyperactivity disorder (ADHD), characterized by age‐inappropriate inattention, hyperactivity, and impulsivity, is a childhood‐onset neurodevelopment disorder which could persist into adulthood.^1^ Inhibition deficits, the core cognitive impairments in ADHD,^2^ are closed to the core symptoms of ADHD^3^ and might be responsible for secondary deficits in other executive functions.^4^ Thus, understanding the mechanism of inhibition would definitely promote our understanding and interpreting the pathogenesis of ADHD.

A core network of inhibition was identified in healthy subjects most prominently in the inferior frontal gyrus, superior frontal gyrus, the supplementary/pre‐supplementary motor area, basal ganglia, and temporal/parietal areas.^5^, ^6^ More recently, studies reported conflicting activation in the cerebellum, cingulate, mesial frontal, and parietotemporal areas in inhibition process of adults with ADHD (aADHD).^7^, ^8^, ^9^ Compelling evidence has confirmed that inhibition impairments involved both brain structural (eg, gray matter volume, GMV) and functional (eg, functional connectivity, FC) dysfunctions in aADHD.^10^ The information provided by the single imaging modality is limited, while multi‐mode could provide much more rich information. The main goal in integrating/fusing studies is to take advantage of the relative strengths of each modality to provide results synergistically for a more comprehensive understanding of brain activity. The joint analysis of multiple brain imaging measures could capture more views and covariations of brain modalities, which was believed to be essential for understanding brain networks and their relationship with human cognition and behavior. However, most studies focused either single modality or a multimodal comparison after individual analysis in each mode, in which cases, the cross‐information among multiple modalities was often missed. In addition, compared with unsupervised fusion, supervised multimodal fusion takes advantage of the prior knowledge, for example, clinical symptoms or cognitive functions, to guide the fusion analysis and can discover more precisely and robustly goal‐directed imaging features.^11^ There have been studies using supervised multimodal fusion approach to look for multimodal biomarkers of schizophrenia^11^ and depression,^12^ but not yet for ADHD.

Inhibition was potentially inheritable characteristics in ADHD.^13^, ^14^ Recently, the *NOS1* gene, encoding neuronal nitric oxide synthase (nNOS), has been suggested to be a candidate gene for aADHD.^15^ The nNOS‐derived nitric oxide (NO) acts as a second messenger downstream of the N‐methyl‐D‐aspartate (NMDA) receptor and interacts with the monoaminergic systems.^16^ Numerous evidence has supported the crucial role of the *NOS1*‐nNOS‐NO pathway in brain development and functions, and in the etiology of neuropsychiatric disorders including ADHD.^17^ In the existing literature, a functional variant, *NOS1* ex1f‐VNTR, has been indicated to be associated with the hyperactive‐impulsive behavior in aADHD.^18^ Imaging genetic exploration showed that the risk short allele (S) of *NOS1* ex1f‐VNTR was associated with abnormal activation of the dorsolateral prefrontal cortex and inferior frontal cortex during the response inhibition tasks.^19^ This functional variant might affect the activation of anterior cingulated cortex or ventral striatum, and finally lead to the impulsivity‐related behavior.^20^, ^21^ In addition to the brain function, the *NOS1* ex1f‐VNTR also potentially influenced the brain structure, such as the white matter microstructure.^22^ However, no efforts to explore the genetic effects of *NOS1* ex1f‐VNTR on the multimodal imaging patterns has been reported, much less the inhibition‐guided fusion analyses.

In our present study, we firstly aim to identify the co‐varying multimodal MRI patterns in aADHD under the guidance of inhibition function, which has been suggested to be a core impairment of ADHD; Secondly, the potential genetic effects of *NOS1* ex1f‐VNTR on inhibition and its related brain alterations were investigated to build a potential “gene‐brain‐behavior” relationship.

## 2 METHODS

### 2.1 Participants

A total of 119 subjects were recruited in the study. Four aADHD and two controls were excluded because they failed to finish the MRI scan. Finally, 69 aADHD and 44 age‐ and sex‐matched healthy controls (HC) were included for analyses in this study. All aADHD were recruited from the outpatient clinics of Peking University Sixth Hospital and HC were recruited by advertisement. All participants underwent the Structured Clinical Interview for Diagnostic and Statistical Manual of Mental Disorders‐Fourth Edition (DSM‐IV) Axis I Disorders (SCID‐I)^23^ by a senior psychiatrist for diagnosis and potential comorbidity. ADHD diagnosis was further confirmed using the Conner's Adult ADHD Diagnostic Interview.^24^ Patients who were taking drugs (2 with OROS‐MPH and 2 with Atomoxetine) were asked to undergo a washout period of at least 48 h before the MRI scan. This study was approved by the Research Ethics Review Board of Peking University Sixth Hospital. Written informed consent was obtained from all participants.

All participants met the following criteria: (1) right‐handed; (2) no history of head trauma with a loss of consciousness; (3) no history of neurological disorders or other severe disease; (4) no current diagnosis of major depressive disorder, schizophrenia, clinically significant panic disorder, bipolar disorder, pervasive developmental disorders, or mental retardation; (5) no excessive head movements (>3.0 mm of translation or 3 degrees of rotation in any direction); and (6) a full‐scale intelligence quotient (FIQ) above 80 (measured by the Wechsler Adult Intelligence Scale, Third Edition). Furthermore, participants with any history of psychiatric disorders were also excluded as healthy controls.

ADHD core symptoms were evaluated by the ADHD Rating Scale‐IV (ADHD RS‐IV).^25^ Eighteen DSM‐IV symptoms (rated on a 4‐point Likert‐type scale ranging from 1 = *never* to 4 = *always*) were summed to yield inattentive, hyperactive/impulsive and total scores.

Inhibition function was assessed by the “*Inhibit”* factor score from Behavior Rating Inventory Executive Function—Adult Version (BRIEF‐A).^26^ The participants were asked to complete 75 items on a 3‐point Likert‐type scale (1 = *never*, 2 = *sometimes*, 3 = *often*) to evaluate their performance in daily life.

### 2.2 MRI data acquisition and parameters for scanning

MRI data were acquired on a GE Signa 3 T Horizon HDx system (General Electric, Milwaukee, WI) at the Centre for Neuroimaging Sciences, Peking University Sixth Hospital. Participants were required to lie in the supine position and remain still and relaxed with their eyes closed but not falling asleep during the resting‐state fMRI scanning. The parameters on the GE scanner were repetition time (TR) = 2000 ms, echo time (TE) = 30 ms, flip angle = 90°, matrix = 64 × 64, field of view (FOV) = 220 mm × 220 mm, 43 axial slices, slice thickness = 3.2 mm, slice gap = 0 mm in resting‐state fMRI. The following parameters were in T1 on GE scanner: TR = 6.7 ms, TE = Min Full, flip angle = 8°, 180 slices, slice thickness = 1.0 mm, slice gap = 0 mm, FOV = 256 mm × 256 mm, and matrix = 256 × 256.

### 2.3 MRI data preprocessing and genotyping

The T1‐weighted structural MRI (sMRI) and resting‐state functional MRI (fMRI) data were analyzed by the Data Processing Assistant for Resting‐State fMRI^27^ (DPARSF, http://rfmri.org/DPARSF) according to standard preprocessing procedures. The GMV was analyzed by voxel based morphometry (VBM) and sMRI data were re‐oriented, segmented into white matter, gray matter, and cerebrospinal fluid using a unified segmentation approach,^28^ normalized into the Montreal Neurological Institute (MNI) space, modulated, and smoothed using a 8 mm Gaussian kernel. fMRI data preprocessing included removing first ten volumes, slice timing correction, head motion correction, spatial normalization to the MNI template, resampling to 3 × 3 × 3 mm^3^, smoothing using a 4 mm Gaussian kernel, temporal band‐pass filtering (0.01 to 0.1 Hz), nuisance signal regression (including 6 head motion parameters, white matter, cerebrospinal fluid, and global signals), and head motion scrubbing.^29^ The FC was analyzed by calculating Pearson correlation coefficients between pairs of node time courses within the whole brain and normalizing to z value using Fisher's transformation.

The peripheral blood genotyping of the *NOS1* ex1f‐VNTR were performed according to the methods described in Rief et al.^30^ We first converted the genotypes to short (16‐27 “GT” repeats, S) and long (28‐36 “GT” repeats, L) alleles followed the methods described in Weber et al.,^18^ then counted three common repeats with relative high frequency at single allele level, including 25‐repeat (25R), 26‐repeat (26R), and 30‐repeat (30R). For example, three genotypes were defined according to the allele accounts of 25R: 25R^+^/25R^+^ indicated the carriers with two 25R, while 25R^+^/25R^−^ as carriers with one 25R, and 25R^−^/25R^−^ as carriers without 25R.

### 2.4 Fusion with reference

**FIGURE 1:**
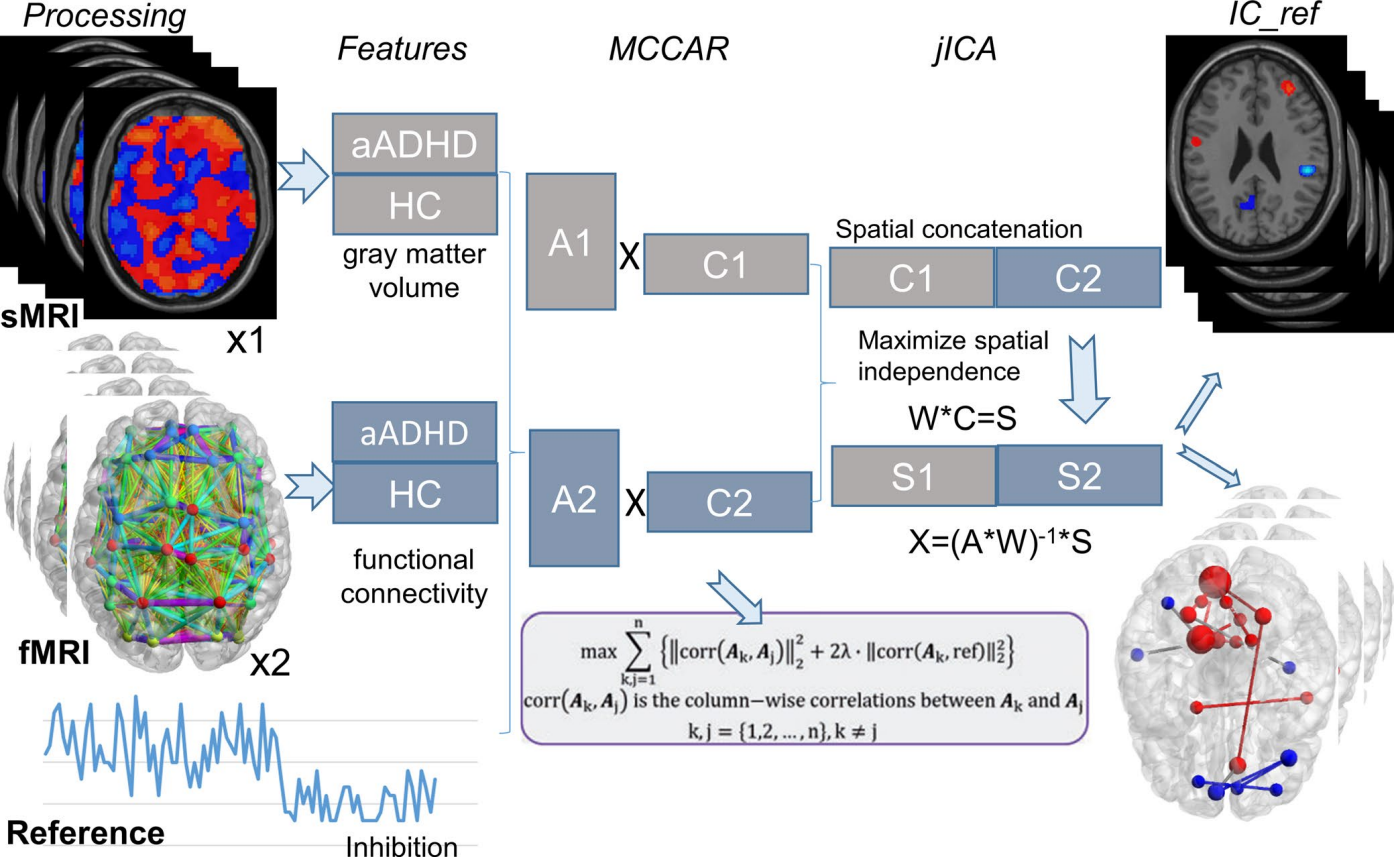
The brief flowchart of the supervised data fusion strategy. This model simultaneously maximizes the inter‐modality covariation and correlations of joint independent component and the prior information (the reference). MCCAR, multisite canonical correlation analysis with reference; jICA, joint independent component, IC_ref, joint independent component related to the reference

Multisite canonical correlation analysis with reference plus joint independent component analysis (MCCAR + jICA) is a supervised, goal‐directed model that enables a priori information (eg, cognitive function and symptoms) as a reference to guide multimodal data fusion. This model can maximize the inter‐modality covariation and precisely identify the co‐varying multimodal component closely related to reference information, which may not be detected by a blind (unsupervised) multimodal fusion approach. In this study, the GMV feature and FC feature were jointly analyzed by “MCCAR + jICA” in which “inhibition” was set as the reference to guide the joint decomposition of this two MRI features. For the code and more information about this method, please refer to the Fusion ICA Toolbox (FIT, https://www.nitrc.org/projects/fit) and the methodology paper.^11^ Thirty components were estimated according to an improved minimum description length criterion.^31^ Two‐sample *t*‐tests were further performed on mixing coefficients of each component for each modality. We aimed to investigate the joint independent component (IC), which is significantly correlated with inhibition, co‐varying among modalities and group discriminative. The brief flowchart of the supervised data fusion strategy could be found in Figure 1.

### 2.5 Analyses of “gene (*NOS1* ex1f‐VNTR)‐cognition (inhibition)” and “gene (*NOS1* ex1f‐VNTR)‐brain (structural/functional patterns)” relationship

The analyses of covariance (ANCOVA) was performed to explore the potential genetic effects of *NOS1* ex1f‐VNTR on inhibition and its related brain structural/functional alterations, while genotypes, ADHD diagnosis and genotype*ADHD diagnosis interaction were entered into the model with gender and age as covariates. When the main genotypic effect and/or genotype*diagnosis interaction effects existed, genetic association was then analyzed in case and control groups separately.

### 2.6 Mediation analysis

Once a joint GMV‐FC fusion pattern was identified to be group discriminative as well as associated with inhibition performance, and inhibition exhibited significant difference in different *NOS1* ex1f‐VNTR genotypes, mediation analysis was further conducted to better delineate the gene‐sMRI/fMRI‐inhibition pathways.

PROCESS macro (http://wwwafhayescom/public/process) in SPSS v.23 was used to conduct these analyses. Bootstrapping was used to estimate the confidence interval (CI) of sampling distributions and the indirect effects of mediators. In this study, bootstrapping sampled from the data 5000 times and provided 95% CI of indirect effects. If the 95% CI does not contain zero, it suggests that the mediating effect is significant at *p* < 0.05 level. In all mediation analysis, age and gender were controlled as covariates.

## 3 RESULTS

### 3.1 Demographic information and Clinical characteristics

Detailed information of the participants recruited in the current study was shown in Table 1. Twelve patients had a history of major depressive disorder and four patients had a history of anxiety disorder. One patient had ever taken osmotic release system methylphenidate (OROS‐MPH) for two months in 2013. Four patients were taking drugs (2 with OROS‐MPH and 2 with Atomoxetine). Age, gender, and FIQ were not significantly different between aADHD and HC. As expected, aADHD were with higher core symptoms than HC, including inattentive, hyperactive/impulsive, and total symptoms. For inhibition function evaluated by the “Inhibit” score of BRIEF‐A, aADHD showed significant higher scores indicating severe deficiency of inhibition function [(16.91 ± 3.21) versus (10.18 ± 2.08), *p* < 0.001].

**TABLE 1:** The demographic and clinical characteristics of all subjects recruited

	aADHD (*N* = 69)	HC (*N* = 44)	*t*/*χ* ^2^ value	*p* value
Age (Mean ± SD)	25.37 ± 4.63	26.24 ± 3.94	−1.03	0.304
Sex, *n* (male/female)	46/23	23/21	2.34	0.126
FIQ (Mean ± SD)	119.53 ± 7.90	120.46 ± 7.23	−0.59	0.556
ADHD subtypes, *n* (%)				
ADHD‐I	11 (15.9)	−	−	−
ADHD‐C	58 (84.1)	−	−	−
ADHD symptoms (Mean ± SD)				
Inattentive	27.11 ± 4.40	13.60 ± 3.46	15.66	<0.001
Hyperactive/impulsive	18.98 ± 5.06	12.40 ± 2.80	8.29	<0.001
Total	46.10 ± 7.82	26.00 ± 5.89	13.25	<0.001
“Inhibit” score (Mean ± SD)	16.91 ± 3.21	10.18 ± 2.08	11.87	<0.001

aADHD, adults with attention‐deficit/hyperactivity disorder; HC, healthy controls; SD, standard deviation; FIQ, full‐scale intelligence quotient; ADHD‐I, prominently inattentive subtype; ADHD‐C, combined subtype.

### 3.2 Inhibition‐guided multimodal co‐varying imaging patterns and their association with ADHD core symptoms and inhibition‐related cognitive functions

**FIGURE 2:**
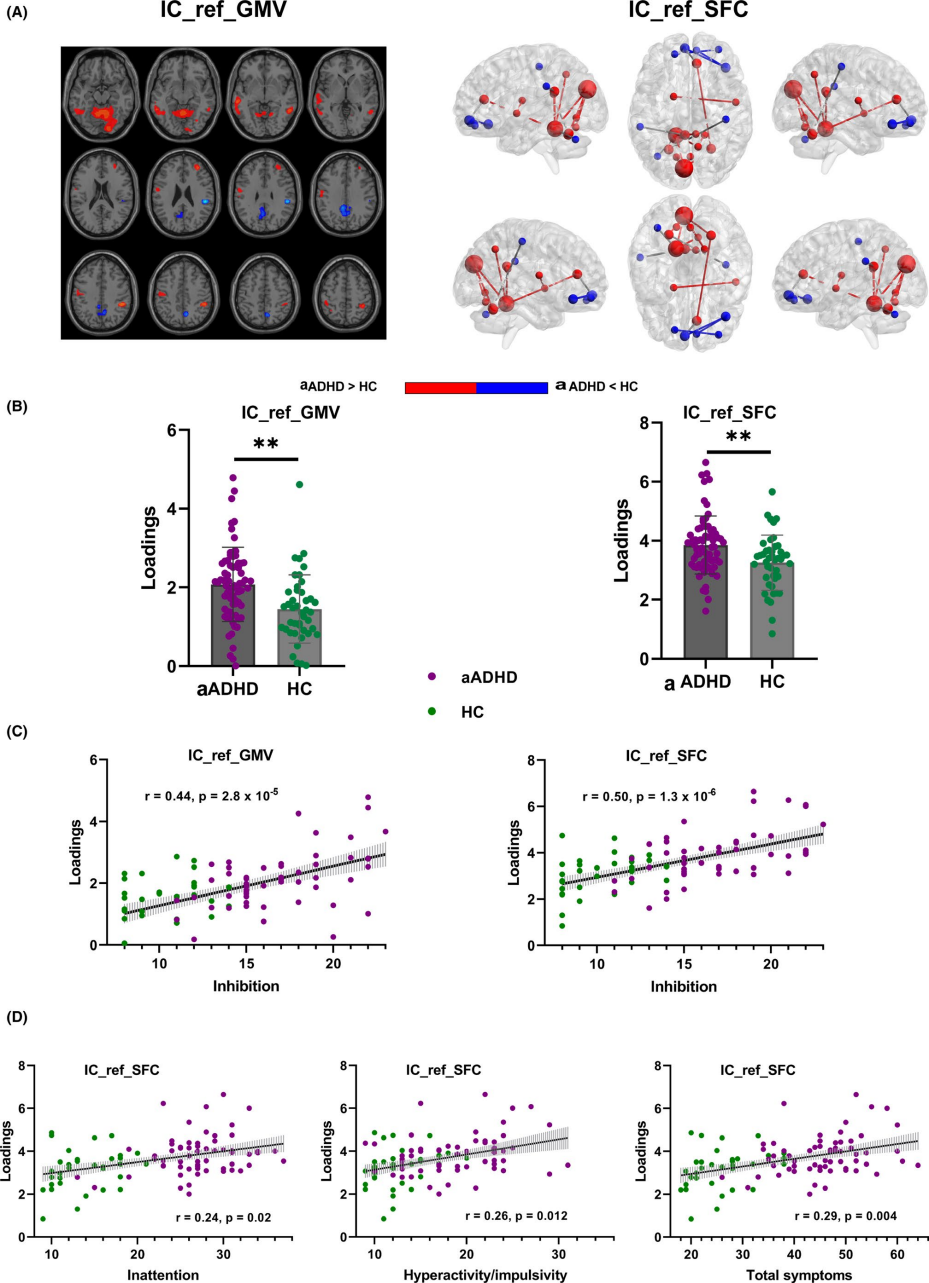
The identified joint independent component. The joint independent component (A) indicated significant group difference (B) and association with inhibition function (C) and ADHD core symptoms (D). IC_ref_GMV, the reference guided joint independent component_gray matter volume; IC_ref_FC, the reference guided joint independent component_functional connectivity; aADHD, adults with attention‐deficit/hyperactivity disorder; HC, healthy controls

The spatial maps of the joint component (denoted as IC_ref, with the same IC order in both structural and functional modalities) were transformed into Z scores, visualized at |Z| > 3 as in Figure 2A (simultaneously cluster size > 200 in GMV). The positive Z‐values indicated higher contribution in aADHD than HC and the negative Z‐values indicated higher contribution in HC than aADHD. The main regions in the IC_ref included cerebellum, superior parietal lobule, inferior orbitofrontal cortex, anterior cingulum, supramarginal gyrus, precentral/postcentral gyrus, and pallidum, which were generally attributed to the inhibition network; inferior temporal gyrus extending to middle temporal gyrus, medial frontal gyrus, precuneus, and posterior cingulum, which mainly within the default mode network (DMN). The more details of the identified GMV regions and FC in the IC_ref were summarized in Table S1.

The joint component was found to be both significantly group discriminating (GMV: *P* = 2.3 × 10^−4†^; FC: *p* = 0.0019^†^; Figure 2B, hereafter† meaning passing FDR correction for multiple comparison) and correlated with inhibition (GMV: *r* = 0.44, *p* = 2.8 × 10^−5†^; FC: *r* = 0.50, *p* = 1.3 × 10^−6†^; Figure 2C) after controlling for age, gender, and diagnosis. Higher “Inhibit” score in aADHD was linked with increased GMV in cerebellum, superior parietal lobule, inferior temporal gyrus extending to middle temporal gyrus, precentral/postcentral gyrus, and middle frontal gyrus; and decreased GMV in supramarginal gyrus, precuneus extending to cingulum and fusiform lobule. Higher “Inhibit” score was associated with higher FC mainly in connectivity within cerebellum, connectivity within inferior orbitofrontal cortex, cerebellum‐postcentral gyrus, posterior cingulum‐supramarginal gyrus, and anterior cingulum‐medial orbitofrontal cortex; and lower FC in cerebellum‐cuneus, cerebellum‐cingulum and cerebellum‐precuneus, etc. These results indicated that reduced and/or compensatory increased GMV/FC of brain regions within the inhibition network, and increased and/or compensatory reduced GMV/FC of brain regions within the DMN were contributed to inhibition deficits in aADHD together.

In addition to “inhibition” function, we also conducted correlation analyses for ADHD core symptoms and other multiple inhibition‐related cognitive domains such as emotional control, shifting, and self‐monitoring which constituted the BRI of BRIEF_A to further identify the reliability of the findings to some extent. As shown in Figure 2D, IC_ref loadings in FC were significantly linked with inattentive (*r* = 0.24, *p* = 0.020^†^), hyperactive/impulsive (*r* = 0.26, *p* = 0.012^†^), and total scores (*r* = 0.29, *p* = 0.004^†^) after controlling for age, gender, and diagnosis. Increased IC_ref loadings in GMV were significantly correlated with higher inattentive (*r* = 0.21, *p* = 0.043) and marginally significantly correlated with total scores (*r* = 0.18, *p* = 0.080) after controlling for age, gender, and diagnosis.

Both GMV loadings and FC loadings showed significantly positive correlation with self‐monitoring and BRI in ADHD patients after controlling for age, gender, and diagnosis. Moreover, GMV loadings were significantly correlated with emotional control (more details see Table S2).

### 3.3 Genetic effects of *NOS1* ex1f‐VNTR genotypes on inhibition and its related brain structural/functional patterns

**FIGURE 3:**
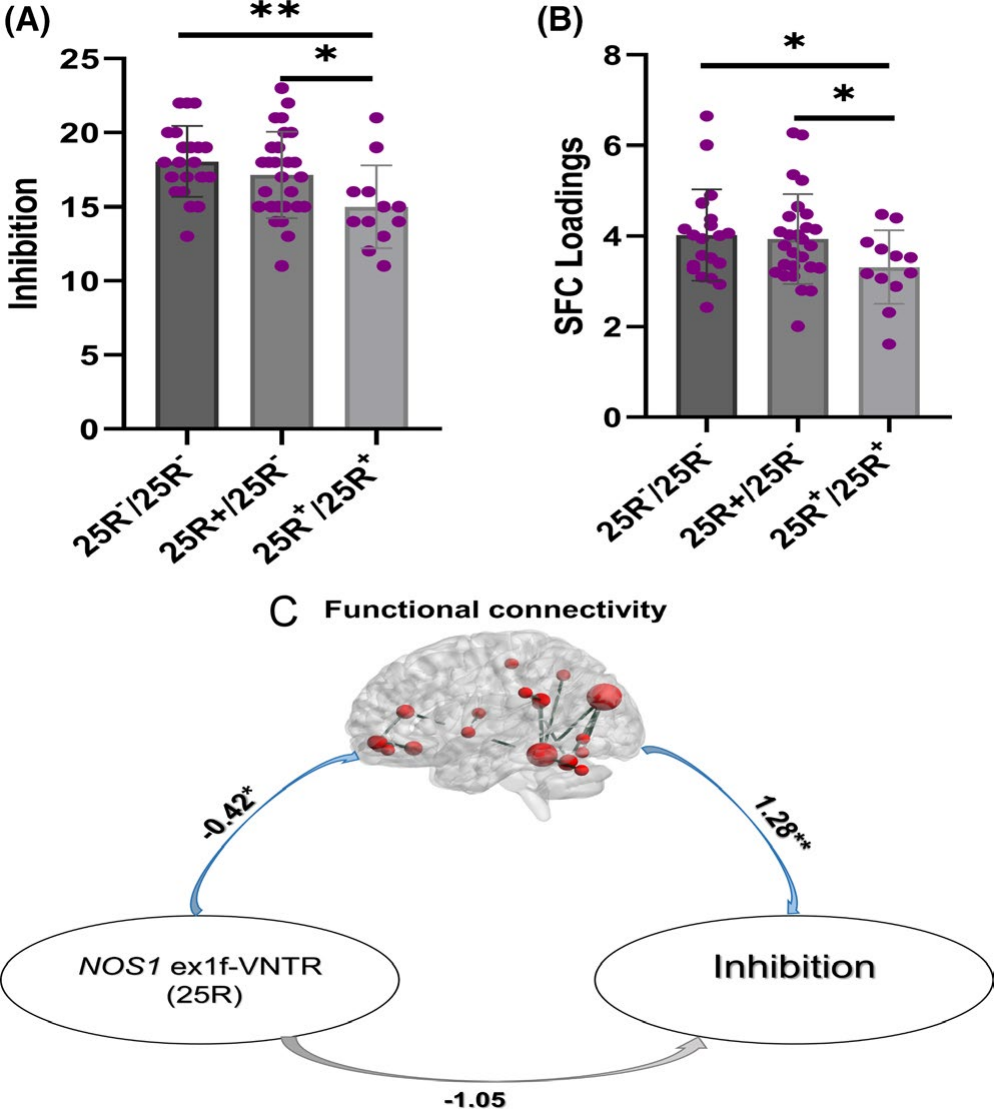
The relationship of *NOS1* ex1f‐VNTR (25R), inhibition, and inhibition‐directed FC in aADHD. Genetic effect of 25R was found on inhibition (A) and inhibition‐directed FC pattern (B), and the FC‐mediated *NOS1* ex1f‐VNTR (25R) and inhibition in aADHD (C). *NOS1* ex1f‐VNTR, nitric oxide synthase1 variable number of tandem repeats in exon 1f; aADHD, adults with attention‐deficit/hyperactivity disorder, FC: functional connectivity

Marginally significant interaction of *NOS1* ex1f‐VNTR genotypes (based on the allele counts of 25R) and ADHD diagnosis was found for inhibition function (*F* = 2.82, *p* = 0.067). When analyzing in ADHD and controls separately, significant association was only found in aADHD (*F* = 3.76, *p* = 0.032). As shown in Figure 3A, post hoc analysis showed that “Inhibit” score in 25R^+^/25R^+^ carriers was significant lower than that in 25R^−^/25R^−^ [(14.63 ± 1.02) versus (18.07 ± 0.75), *p* = 0.009] or 25R^+^/25R^−^ carriers [(14.63 ± 1.02) versus (17.20 ± 0.66), *p* = 0.042]. No significant and/or marginally significant genetic effects of other *NOS1* ex1f‐VNTR genotypes (S/L, 26R, 30R) on inhibition function were found.

For the imaging features, marginal main genetic effect of 25R was found on FC (*F* = 2.97, *p* = 0.058). When analyzing separately, marginal significant association only existed for aADHD (*F* = 2.62, *p* = 0.085). Post hoc analysis showed that FC in 25R^+^/25R^+^ carriers was significant lower than that in *NOS1* ex1f‐VNTR with 25R^−^/25R^−^ [(3.14 ± 0.36) versus (4.11 ± 0.27), *p* = 0.037] and 25R^+^/25R^−^ [(3.14 ± 0.36) versus (4.03 ± 0.24), *p* = 0.048] (see Figure 3B). No significant and/or marginally significant association was found for GMV.

**FIGURE 4:**
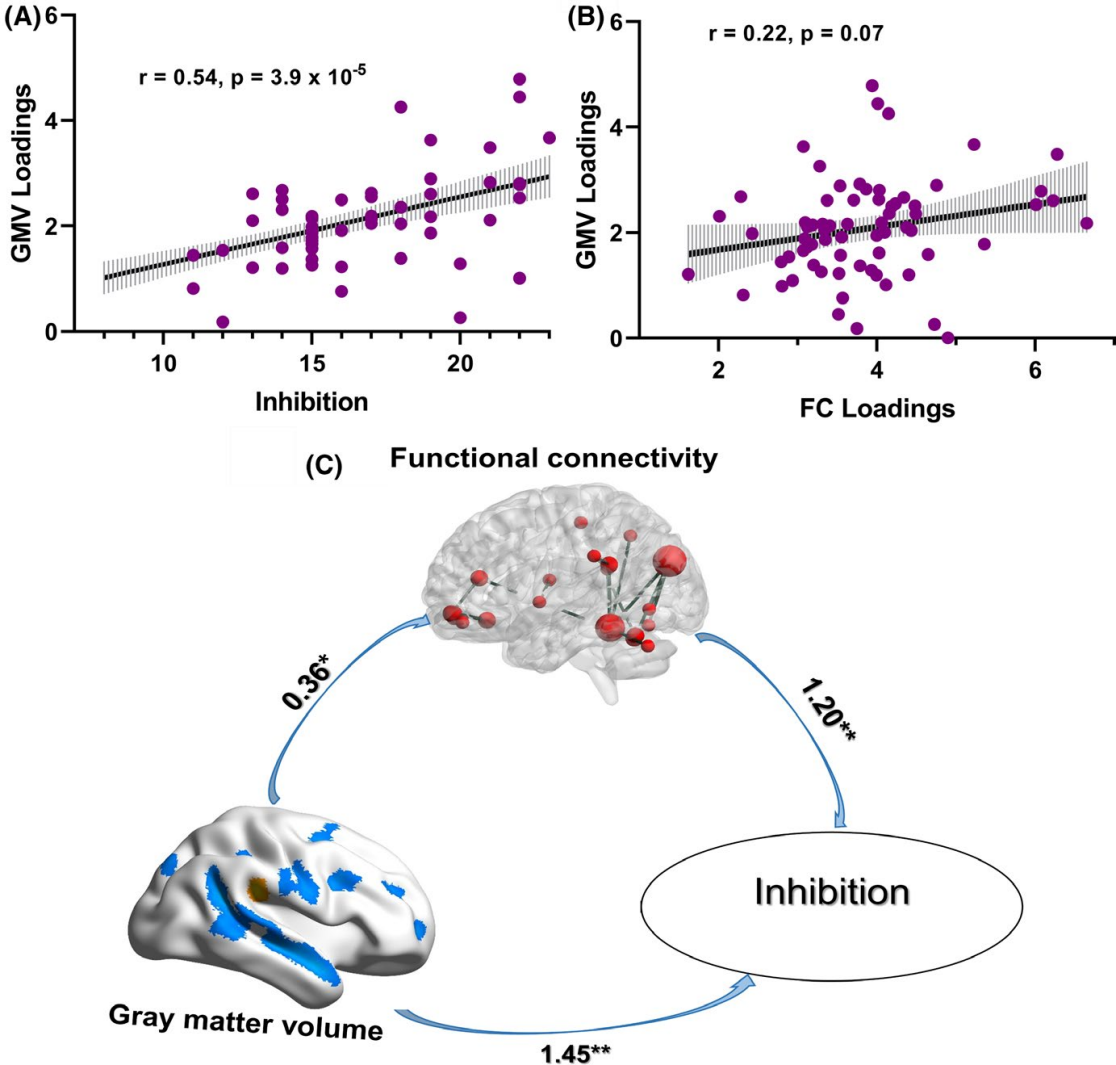
The relationship of inhibition, inhibition‐directed GMV, and inhibition‐directed FC in aADHD. GMV could affect inhibition not only directly, but also through the mediation effect of the FC in aADHD. aADHD, adults with attention‐deficit/hyperactivity disorder; GMV, gray matter volume; FC, functional connectivity

Besides, GMV was significantly correlated with inhibition (*r* = 0.44, *p* = 2.8 × 10^−5†^) and marginally significantly correlated with FC (*r* = 0.17, *p* = 0.078) after controlling for age, gender, and diagnosis. Specifically, the correlations between inhibition and GMV loadings were significant in aADHD but not in HC (aADHD: *r* = 0.54, *p* = 3.9 × 10^−5†^, Figure 4A; HC: *r* = 0.28, *p* = 0.12) after controlling for age and gender. The correlations between FC and GMV loadings were marginally significant in aADHD but not in HC (aADHD: *r* = 0.22, *p* = 0.070, Figure 4B; HC: *r* = 0.10, *p* = 0.54) after controlling for age and gender.

### 3.4 Mediation effects

Based on the above findings of “gene‐cognition,” “gene‐brain,” and “brain‐cognition” relationship, the following mediation analyses were conducted among the *NOS1* ex1f‐VNTR (25R) genotypes, FC, GMV and inhibition in aADHD.

In the mediation model of NOS1 ex1f‐VNTR (25R) genotypes, FC, and inhibition, the path from the *NOS1* ex1f‐VNTR (25R) genotypes to FC was significant [B = −0.42 (SE = 0.22), *p* = 0.048], and the path from FC to inhibition was significant [B = 1.28 (SE = 0.39), *p* = 0.002], but the path from the *NOS1* ex1f‐VNTR (25R) genotypes to inhibition did not reach statistical significance. Figure 3C indicated a significant indirect effect of *NOS1* ex1f‐VNTR on the inhibition via FC component [effect size = −0.54 (SE = 0.29), 95% CI = −1.16 to −0.01]. However, the direct effect from *NOS1* ex1f‐VNTR to inhibition after the addition of FC to the model was not significant [effect size = −1.04 (SE = 0.58), 95% CI = −2.21 to −0.11], which suggested that *NOS1* ex1f‐VNTR affected inhibition completely through the mediation effect of the aforementioned FC (*NOS1* ^→FC→^Inhibition), not directly.

In the mediation model of GMV, FC, and inhibition, the path from the GMV to FC was significant [B = 0.36 (SE = 0.16), *p* = 0.029], the path from FC to inhibition was significant [B = 1.20 (SE = 0.34), *p* = 0.001], and the path from the GMV to inhibition was significant [B = 1.89 (SE = 0.42), *p* < 0.001]. Figure 4C indicated a significant indirect effect of GMV on the inhibition via FC component [effect size = 0.43 (SE = 0.23), 95% CI = 0.12 to 1.00]. The direct effect from GMV to inhibition after the addition of FC to the model was also significant [effect size = 1.45 (SE = 0.39), 95% CI = 0.66 to 2.24], which suggested that GMV could affect inhibition not only directly, but also through the mediation effect of the aforementioned FC (GMV^→FC→^Inhibition).

## 4 DISCUSSION

This study provided several new insights into the relationships between inhibition performance, related brain structural/functional alterations, and *NOS1* ex1f‐VNTR genotypes in aADHD. First, we attempt to reveal how abnormally structural and functional neuroimaging features jointly contribute to inhibition impairment in aADHD. To the best of our knowledge, this is the first study to explore the inhibition‐guided multimodal brain imaging fusion in aADHD. Second, we explored how genetic variants and brain structural/functional alterations worked relatedly to cause inhibition deficit by constructing a potential “gene‐brain‐cognition” pathway. Our findings indicated that both the inhibition network and the DMN were involved in inhibition deficits for aADHD. In addition, the FC might mediate the relationship of the functional genetic variants, *NOS1* ex1f‐VNTR and inhibition, as well as the relationship of the GMV and inhibition in aADHD.

Recently, more and more studies were designed to explore brain morphometric and functional abnormalities and alterations in aADHD. aADHD‐related morphometric alterations and abnormal activation emerged in large‐scale networks, predominantly located within the frontoparietal, visual, dorsal attention, and default mode networks and so on.^32^, ^33^, ^34^, ^35^ In analyses limited to inhibition tasks, aADHD‐related abnormal activation included several frontal regions bilaterally, parieto‐occipital cortex, the right superior temporal gyrus, the left inferior occipital gyrus, and the right thalamus.^32^ Due to the limitation of analytical methods, there were a few researches focusing on structural anomalies related to inhibition and the results were inconsistent.^10^

In the study, a supervised, goal‐directed fusion model was used to explore the underlying structure‐function fusion pathogenesis of inhibition in aADHD. “Inhibit” score was used as a specific prior reference to guide the fusion in this study. Results showed that the gradual addition of GMV and FC variables could improve the interpretation level of inhibition variability (more details see the supplementary results and Table S3), indicating multi‐modalities provided more enriched imaging information than unimodal pattern. In addition, it was able to extract more sensitive structure‐function dysfunctions associated with the inhibition itself than unsupervised fusion approaches.^11^, ^36^, ^37^ Specifically, the modality‐common co‐varying regions including the cerebellum, precuneus, primary motor area, supramarginal area, cingulate cortex and prefrontal areas (both detected in GMV and FC), and the modality‐specific regions including superior parietal lobule, middle, and inferior temporal areas (only detected in GMV) and basal ganglia (only detected in FC). This indicated a varied but well‐defined set of brain areas that have usually been associated with inhibition in ADHD. According to the prominent opinions regarding the neural substrates of calling off an ongoing response, cortical areas involved in the inhibition network, like inferior frontal cortex and pre‐supplementary motor area, send a stop command to basal ganglia structures, increase inhibitory signals from the globus pallidus, and thus inhibit the output from the basal ganglia.^38^ Anterior cingulate activation was considered to be error‐related in a conflict situation, or even a more general role as a conflict detector, independent of whether an error occurred.^39^ The parietal lobes are thought to reflect reorientation of attention and the maintenance of task sets during inhibition process.^6^, ^40^ The lateral cerebellum projects to the motor area through the thalamus and to the prefrontal cortex, which may contribute to both the preparation and inhibition of movement.^9^, ^41^ In addition to abnormal alternations of above regions within the inhibition network, failed suppression of irrelevant network (the DMN) was key for inhibition impairments.^13^, ^42^, ^43^ Behavioral inhibition depends on the integrity of the inhibition network and the DMN.

Not only reduced GMV and FC within the inhibition network, but also increased GMV and FC within the DMN were involved in inhibition deficits in aADHD. This implicated the abnormalities observed in ADHD patients affected not only isolated brain structure regions but also the functional connectivity between these brain regions, thus providing a structure‐function fusion neural mechanism for inhibition deficits in aADHD. A central assumption of neuroscience is that brain structure could predict and/or is linked to brain function.^44^ Accordingly, we have also conducted mediation analyses for the potential “GMV^→FC→^Inhibition” model. As expected, the GMV of the abovementioned regions could play a role not only directly but also through the mediation of FC in inhibition impairment. As far as we know, this is the first attempt to guide multimodal imaging data fusion with cognitive scores as a reference to seek potential multimodal neural markers of inhibition deficits in ADHD.

In our present study, we have found a marginal genetic association of *NOS1* ex1f‐VNTR with impaired inhibition function in aADHD, which was consistent with previous report in some extent.^45^ However, we should note that the potentially risk allele indicated in our present study was the “25R,” but not the short allele (S) or the “21R” reported previously in the Caucasian populations.^18^ One potential reason might be the ethnic difference. When comparing the allelic distribution, we could found that the most common alleles in Chinese Han subjects were 25R, 26R, and 30R, while that in Caucasian subjects were 20R, 25R, and 31R.^18^ Similarly, the negative association for the short allele (S) in our study may be due to the potentially different definition of S or L allele in Chinese Han subjects which needs future functional analyses. Further imaging genetic analyses indicated that this gene‐cognition association was potentially mediated by the brain resting‐state functional alteration in inhibition and default mode networks. In a previous report, significant decreased activity in dorsolateral prefrontal cortex and inferior frontal cortex was found for risk short allele carriers of *NOS1* ex1f‐VNTR during inhibition process.^19^ Reif et al. speculated that the hypoactivation in prefrontal cortex in risk allele carriers, leading to compromised inhibition processes, might be due to decreased NO production as a consequence of reduced expression of *NOS1* gene.^46^ Besides dorsolateral prefrontal cortex and inferior frontal cortex, previous studies reported abnormal anterior cingulate cortex, parietal cortex and basal ganglia activation in risk *NOS1* allele carriers,^20^, ^21^, ^30^, ^46^ which were all the central regions for inhibition. Consistent with these results, our results showed that *NOS1* ex1f‐VNTR affected inhibition through the mediation of FC of the inhibition network and DMN.

There are some limitations in current study. Firstly, the sample size in genetic subgroup is relatively small, which might lead to marginal findings in genetic association analyses. In addition, we did not perform analyses for males and females separately, but just set as covariate. A larger sample size may lead to more robust results and help us to illustrate the potential gender‐specific relationship in the future. Secondly, fusion modal included only two modalities of GMV and FC in this study. Inhibition may also be affected by white matter integrity, local spontaneous brain activity, or cerebral blood flow. The fusion of these modalities would provide more informative knowledge of inhibition and could be studied in the future.

## 5 CONCLUSIONS

Our results showed that reduced GMV and FC in the inhibition network and increased GMV and FC in the DMN were jointly responsible for inhibition deficits in aADHD. More importantly, both the functional *NOS1* ex1f‐VNTR variant and the GMV might affect inhibition through the mediation of the abnormal FC of these brain regions, identified a potential gene‐brain‐cognition pathway for inhibition in aADHD. This multimodal fusion method may be helpful in searching for meaningful biological markers and cross‐information links in multiple neuroimaging modalities. Integration of multimodal brain imaging and individual‐level genetic data delineates a new way for elucidating the pathophysiology and provides potential biomarkers of inhibition impairments.

## CONFLICT OF INTEREST

All authors reported no potential conflicts of interest.

## Supporting information

Supplementary Material

## Data Availability

The raw data required to reproduce these findings cannot be shared at this time as the data also forms part of an ongoing study.
