# Supplementary material for: Inhibition‐directed multimodal imaging fusion patterns in adults with ADHD and its potential underlying “gene‐brain‐cognition” relationship
Source: CNS Neurosci Ther. 2021 Mar 16;27(6):664–73. doi: 10.1111/cns.13625 (PMC8111492; doi:10.1111/cns.13625)
Supplement: Supplementary file 1 — Supplementary Material [file CNS-27-664-s001.docx]

Supplementary Information

**Inhibition-directed multimodal imaging fusion patterns in adults with ADHD and its potential underlying ‘gene-brain-cognition’ relationship**

**Supplementary methods**

**Statistical analysis**

Kolmogorov-Smirnov test for normality was used to assess data distribution in the study. If it exhibited a normal distribution, the data were described by means and standard deviation and compared by independent samples *t*-test. If it did not exhibit a normal distribution, the data were described by interquartile range and compared by Kolmogorov-Smirnov test. Chi-square test were used to compare the categorical data.

**Supplementary results**

**The association of inhibition-guided multimodal co-varying imaging patterns with inhibition and ADHD core symptoms**

Specifically, the correlations between inhibition and GMV loadings were significant in aADHD but not in HC (aADHD: *r* = 0.54, *P* = 3.9 × 10^-5†^; HC: *r* = 0.28, *P* = 0.12), and the correlations between inhibition and FC loadings were significant in both aADHD and HC (aADHD: *r* = 0.53, *P*= 5.5 × 10^-5†^, HC: *r* = 0.39, *P*= 0.028) after controlling age and gender.

Specifically, increased IC_ref loadings in FC were significantly linked with higher inattentive (*r* = 0.26, *P* = 0.045), hyperactive/impulsive (*r* = 0.26, *P* = 0.040) and total scores (*r* = 0.32, *P* = 0.013^†^) in aADHD after controlling for age and gender. Not such significant correlations between FC loadings and ADHD core symptoms were observed in HC. However, no significant correlations between GMV loadings and ADHD symptoms were observed in aADHD or HC.

**Hierarchical multiple regression model of GMV and FC on inhibition**

Hierarchical multiple regression was performed to analyze whether the gradual addition of GMV and FC variables could improve the interpretation level of inhibition variability in aADHD after controlling for the influence of gender and age.

When only the GMV variable was added, the R^2^ value increased by 0.293 (model 2, *F* = 20.47, *P* < 0.001). When the FC variable continued to be added, the *R^2^* value was 0.148 higher than that when the GMV variable was included only (model 3, *R^2^* = 0.447, adjusted *R^2^* = 0.440, *F* = 9.68, *P*< 0.001). The final model included two variables, namely GMV and FC, which were both statistical significance (GMV, *P*= 0.001; FC: *P* = 0.001) (**Table S3)**.

**Table S1A** Anatomical information of the identified joint component in GMV component

| **Area** | **Cluster size** | **x** | **y** | **z** | **Z-Value** |
| --- | --- | --- | --- | --- | --- |
| **aADHD > HC** | | | | | |
| Cerebelum | 26463 | -39 | -57 | -51 | 9.9146 |
| Parietal_Sup_R | 1619 | 46.5 | -39 | 42 | 6.1075 |
| Temporal_Inf_L extending to Temporal_Mid_L | 2851 | -43.5 | -27 | -24 | 5.2502 |
| Temporal_Inf_L extending to Temporal_Mid_L | 482 | 55.5 | -49.5 | -4.5 | 5.0723 |
| Precentral_L | 267 | -18 | -13.5 | 64.5 | 4.8046 |
| Parietal_Sup_L | 702 | -33 | -52.5 | 61.5 | 4.698 |
| Frontal_Mid_R | 481 | 31.5 | 43.5 | 25.5 | 4.6571 |
| Postcentral_L | 696 | -55.5 | -4.5 | 25.5 | 4.0464 |
| Temporal_Inf_R | 367 | 45 | -28.5 | -24 | 3.9017 |
| **aADHD < HC** | | | | | |
| SupraMarginal_R | 451 | 49.5 | -31.5 | 27 | -6.4373 |
| Precuneus extending to cingulum | 1855 | 7.5 | -61.5 | 46.5 | -4.9556 |
| Fusiform_L | 218 | -33 | -9 | -30 | -4.4404 |

GMV, gray matter volume; aADHD, adults with attention-deficit/hyperactivity disorder; HC, healthy controls.

**Table S1B** Anatomical information of the identified joint component in FC component

| **Region A** | **Region B** | **Z-value** |
| --- | --- | --- |
| **aADHD > HC** |  |  |
| Cerebelum_Crus1_L | Cerebelum_4_5_L | 3.654611 |
| Frontal_Sup_Orb_L | Frontal_Mid_Orb_R | 3.423418 |
| Postcentral_R | Cerebelum_6_L | 3.214234 |
| Cingulum_Post_L | SupraMarginal_L | 3.207485 |
| Frontal_Inf_Orb_R | Frontal_Med_Orb_L | 3.059075 |
| Frontal_Med_Orb_L | Cingulum_Ant_R | 3.054314 |
| Frontal_Inf_Orb_R | Frontal_Med_Orb_R | 3.01935 |
| **aADHD < HC** |  |  |
| Cuneus_L | Cerebelum_4_5_R | -4.05433 |
| Cuneus_L | Cerebelum_4_5_L | -3.83942 |
| Cuneus_L | Cerebelum_7_L | -3.5861 |
| Cingulum_Ant_R | Cerebelum_6_R | -3.44751 |
| Cingulum_Post_L | Cerebelum_4_6_L | -3.27003 |
| Cuneus_L | Cerebelum_6_R | -3.25711 |
| Cingulum_Post_R | Cerebelum_4_5_L | -3.21358 |
| Precuneus_R | Cerebelum_4_5_L | -3.20936 |
| Cuneus_L | Lingual_L | -3.17012 |
| Rolandic_Oper_R | Pallidum_L | -3.15714 |
| Cuneus_L | Vermis_6 | -3.04111 |

FC: functional connectivity; aADHD, adults with attention-deficit/hyperactivity disorder; HC, healthy controls.

**Table S2** The correlations of inhibition-directed GMV and FC loadings with executive functions

| **Executive functions** | **GMV** | | | | | | **FC** | | | | | |
| --- | --- | --- | --- | --- | --- | --- | --- | --- | --- | --- | --- | --- |
|  | **aADHD + HC** | | **aADHD** | | **HC** | | **aADHD + HC** | | **aADHD** | | **HC** | |
|  | ***r*** | ***P*** | ***r*** | ***P*** | ***r*** | ***P*** | ***r*** | ***P*** | ***r*** | ***P*** | ***r*** | ***P*** |
| **Shifting** | 0.13 | 0.239 | 0.10 | 0.49 | 0.21 | 0.254 | 0.06 | 0.609 | 0.02 | 0.898 | 0.28 | 0.128 |
| **Emotional control** | 0.36 | 0.001 | 0.47 | 0.001 | 0.21 | 0.247 | 0.174 | 0.113 | 0.16 | 0.273 | 0.19 | 0.298 |
| **Self-monitoring** | 0.23 | 0.033 | 0.36 | 0.010 | 0.17 | 0.357 | 0.28 | 0.009 | 0.33 | 0.020 | -0.06 | 0.747 |
| **BRI** | 0.38 | <0.001 | 0.49 | <0.001 | 0.28 | 0.120 | 0.31 | 0.005 | 0.31 | 0.027 | 0.20 | 0.278 |

BRI, behavioral regulation index; GMV, gray matter volume; FC: functional connectivity; aADHD, adults with attention-deficit/hyperactivity disorder; HC, healthy controls.

**Table S3** Hierarchical multiple regression of GMV and FC on inhibition in aADHD

| **Model** | **Variable** | **Standardized Coefficients** | ***t* Value** | ***P*_1_Value** | ***R^2^*** | ***R^2^* change**  **(*ΔR^2^*)** | ***P*_2_Value** |
| --- | --- | --- | --- | --- | --- | --- | --- |
| **Model 1** | **constant** | − | 5.38 | < 0.001 | 0.005 | 0.005 | 0.880 |
|  | **gender** | -0.007 | -.05 | 0.962 |  |  |  |
|  | **age** | 0.071 | .51 | 0.616 |  |  |  |
| **Model 2** | **constant** | − | 3.66 | 0.001 | 0.298 | 0.293 | 0.001 |
|  | **gender** | -0.090 | -.74 | 0.462 |  |  |  |
|  | **age** | 0.170 | 1.40 | 0.168 |  |  |  |
|  | **GMV** | 0.556 | 4.53 | < 0.001 |  |  |  |
| **Model 3** | **constant** | − | 2.89 | 0.006 | 0.447 | 0.148 | < 0.001 |
|  | **gender** | -0.065 | -0.60 | 0.553 |  |  |  |
|  | **age** | 0.106 | 0.96 | 0.344 |  |  |  |
|  | **GMV** | 0.429 | 3.70 | 0.001 |  |  |  |
|  | **FC** | 0.407 | 3.59 | 0.001 |  |  |  |

*P*_1_, *P* value of coefficients in regression model; *P_2_*, *P* value of ANOVA in regression model; GMV, gray matter volume; FC: functional connectivity; aADHD, adults with attention-deficit/hyperactivity disorder.
